# Reply: Sex Differences in the Impact of Exercise Volume on Subclinical Coronary Atherosclerosis: A Meta-Analysis

**DOI:** 10.1016/j.jacadv.2025.102252

**Published:** 2025-10-17

**Authors:** Ahmed Abdelaziz, Martha Gulati, Michel D. Shapiro, Carl J. Lavie, Leandro Slipczuk

**Affiliations:** a Medical Research Group of Egypt (MRGE), Negida Academy, Arlington, Massachusetts, USA; b Faculty of Medicine, Al-Azhar University, Cairo, Egypt; c The Barbra Streisand Women’s Heart Center, Smidt Heart Institute, Cedars-Sinai Medical Center, Los Angeles, California, USA; d Baim Institute for Clinical Research, Boston, Massachusetts, USA; e Center for Prevention of Cardiovascular Disease, Section on Cardiovascular Medicine, Wake Forest University School of Medicine, Winston-Salem, North Carolina, USA; f John Ochsner Heart and Vascular Institute, Ochsner Clinical School-the UQ School of Medicine, New Orleans, Louisiana, USA; g Montefiore Health System/Albert Einstein College of Medicine, Bronx, New York, USA

We thank Prof Brophy for his interest in our recent study in *JACC: Advances*^1^ as well as the opportunity to respond to their criticisms.

Following the protocol registration, emerging evidence by Ji et al.^2^ from a large prospective cohort, suggested clinically meaningful sex differences in the cardiovascular benefits from equivalent doses of physical activity. In light of these findings and the biological plausibility of sex-specific exercise responses and plaque phenotypes, we amended our analysis plan, before evaluating sex effects in our data set, to include sex-stratified analyses for the association between physical activity and incident subclinical coronary atherosclerosis. We reported the stratified results alongside the overall analysis and interpreted the sex-specific findings as hypothesis-generating.^1^

Male athletes with high-volume exercise (>3,000 metabolic equivalent of task-min/wk) had significantly higher coronary artery calcium (CAC) scores compared with male nonathletes (MD: 31.6; 95% CI: 10.7-52.6; *P* < 0.001), whereas no significant difference was observed for moderate-volume exercise (MD: 1.2; 95% CI: –24.7-27.1; *P* = 0.93). We acknowledge Prof. Brophy’s point regarding interaction testing. However, our intent was not to evaluate a pooled interaction effect but rather to report subgroup-specific estimates, consistent with prior studies by Pavlovic et al.^3^ and Defina et al.^4^ For this reason, we placed emphasis on the separate subgroup findings rather than the overall pooled estimate. Consistent with Prof Brophy’s suggestion, we also calculated the “standardized mean difference (Cohen’s d)”, which confirmed the consistency of our findings.^1^ We recognize that CAC distributions were right-skewed across studies, reflecting heterogeneity in the inclusion criteria. For example, Defina et al^4^ included participants with higher baseline values of CAC compared with other cohorts. These differences underscore the limitations of the available evidence and the need for prospective studies with standardized protocols to validate and extend our observations.^1^

In summary, our analyses suggest the possibility of sex-dependent associations between high-volume exercise and subclinical atherosclerosis, but we view these findings as hypothesis-generating rather than definitive. Clarifying this relationship will require large, prospective studies with standardized measures of coronary plaque and sex-stratified analyses. We welcome continued dialogue on this topic and share Prof. Brophy’s view that more rigorous evidence is needed before firm conclusions can be drawn.
